# Non-immersive virtual environments for the treatment of hoarding disorder: a preliminary randomized controlled trial based on a non-clinical sample

**DOI:** 10.1186/s40359-025-03446-w

**Published:** 2025-09-29

**Authors:** Susanna Pardini, Silvia Olivetto, Massimiliano Martinelli, Caterina Novara

**Affiliations:** 1 Department of General Psychology, University of Padua, Via Venezia 8, Padova, 35131 Italy; 2 Digital Health Research, Centre for Digital Health & Wellbeing, Fondazione Bruno Kessler, Trento, Italy

**Keywords:** Hoarding disorder, Virtual reality based therapy, Preliminary study, Non-immersive virtual scenarios

## Abstract

**Purpose:**

Hoarding Disorder (HD) is characterized by persistent difficulty discarding possessions, leading to excessive accumulation and functional impairment. Traditional treatments, based on cognitive-behavioral therapy (CBT), emphasize motivational enhancement, cognitive restructuring, and exposure-based interventions. However, these methods face challenges, including heightened distress during in vivo exposure and avoidance tendencies. Virtual Reality (VR) has emerged as a promising adjunctive tool, offering immersive, controlled environments for gradual exposure. The current preliminary study investigates the efficacy of non-immersive VR, defined as a computer-generated environment accessed via a standard screen without head mounted displays or motion tracking, in facilitating the discarding of personal belongings compared to imaginative exposure.

**Method:**

Eighty participants from non-clinical convenience sample (mean age = 25.98, SD = 9.84) were randomly assigned to Virtual Reality Exposure (VRe) or Imagination Exposure (Ie). A non-immersive VR environment was created using Blender and GODOT software, simulating a non-immersive virtual home where participants interacted with representations of their objects. Psychological constructs, including state anxiety and positive and negative feelings, were measured using validated self-report tools (STAI-Y1, PANAS). Behavioral outcomes focused on object discarding frequency during exposure and in vivo sessions.

**Results:**

Results demonstrated a significantly higher proportion of successful discarding in the VR group compared to the imagination group during the experimental exposure session (87.5% vs. 50%; *p* < .001).

**Conclusion:**

VR’s realistic sensory-motor engagement may help circumvent cognitive and emotional barriers to exposure tasks, increasing the ecological validity of these techniques. This preliminary study indicates that non-immersive VR may play a role in bridging the gap between imaginative-based exposure therapy and in vivo HD, that is, real-life exposure to discarding personal items. Although replication in clinical populations and long-term outcome data will be needed, non-immersive VR may provide a cheap, broadly available tool to improve treatment engagement and efficacy. Therefore, future research should analyze the relative advantages of immersive-type VR systems and improve assessment instruments to capture subtle changes over time in psychological constructs. This study was conducted according to the Declaration of Helsinki and was approved by the Ethics Committee of Psychological Research (Area 17), University of Padova (approval number: DD0A28FFB2B4C63CFED6CC9BACC7A530).

**Trial registration:**

ClinicalTrials.gov identifier NCT07160543. Retrospectively registered. Approved and published 5 September 2025.

**Supplementary Information:**

The online version contains supplementary material available at 10.1186/s40359-025-03446-w.

## Introduction

Hoarding Disorder (HD) is primarily characterized by difficulties in discarding objects, resulting in excessive clutter and accumulation that compromise the functionality of living spaces [1, 2]. The estimated prevalence of HD in the general population ranges from 2% to 6% [3, 4]. A multi-phase treatment protocol has been developed within the cognitive-behavioral framework to address HD symptoms [5]. The key components of this approach include enhancing treatment motivation, fostering functional decision-making and problem-solving skills—particularly in determining which items to discard and how to organize what remains—and employing cognitive restructuring and exposure-based interventions, both in imagination and in vivo [6]. While some studies have demonstrated statistically significant reductions in symptoms following this treatment [7], others suggest that these improvements may not always translate into clinically meaningful changes, particularly when evaluated against standardized cut-off scores [8]. CBT for HD achieves clinically significant improvement in only 24–43% of cases, leaving up to two-thirds of individuals with persistent symptoms [8, 9]. Furthermore, a substantial proportion of individuals with HD—up to 50%—either refuse CBT or discontinue treatment early, likely due to its focus on decluttering, which is often perceived as highly distressing [6]. This highlights the need for more effective and acceptable interventions aimed at reducing the emotional challenges associated with discarding [10]. To enhance treatment outcomes, some researchers have introduced Virtual Reality (VR) to augment motivational and exposure-based interventions [11–13]. VR offers an innovative method for addressing the complexities of HD [14–15]. A notable advantage of VR is its ability to create controlled, immersive environments where patients can interact with their possessions safely, allowing them to confront their fears of discarding without the immediate stress of real-world consequences [16]. However, immersive VR typically requires expensive equipment such as head-mounted displays and motion-tracking systems, limiting its accessibility in many clinical settings. In contrast, non-immersive VR refers to interactive, computer-generated environments displayed on a standard screen, without the use of headsets or motion sensors. Users navigate and interact with the virtual space using conventional input devices like a mouse and keyboard. Although it does not offer full sensory immersion, non-immersive VR retains key elements of engagement and realism, making it a practical and scalable option for simulating exposure tasks in therapy e.g., [17]. Unlike physical discarding in the real world, VR allows for repeated object disposal, enabling gradual desensitization. Essentially, by continuously rehearsing the act of discarding virtual items in VR, individuals may find real-life discarding less distressing. This virtual practice can act as a bridge, making it easier to transition to discarding objects in the real world [16]. This technology enables therapists to simulate real-life scenarios, allowing patients to practice decision-making regarding their belongings in a supportive, non-threatening environment [16]. Despite the growing body of evidence supporting immersive VR for psychological treatments, the potential of non-immersive VR remains relatively understudied. Non-immersive VR provides a less resource-intensive alternative, offering similar interactive environments without requiring expensive headsets or high-end systems. This distinction is critical, as many individuals and clinical settings may lack the financial or technological means to access fully immersive VR systems. Exploring non-immersive VR addresses this gap by investigating its efficacy as a practical and scalable solution for HD interventions. By reducing the cost and complexity of VR systems, non-immersive VR has the potential to make innovative treatment options accessible to a broader population, including those in underserved or resource-limited environments. Evidence suggests that VR can effectively implement interventions for various psychological and psychiatric disorders [18]. One promising approach to enhancing HD treatment involves modifying exposure to activating stimuli, particularly the act of discarding objects. Gradual exposure, first in imagination and then in vivo, has been shown to help patients manage the negative emotions associated with this behavior [19]. However, both methods have limitations, including difficulty imagining the act of discarding, heightened emotional distress during in vivo exposure, and the potential for treatment avoidance [20]. Research indicates that virtual environments can provide a safe and effective means of gradually exposing individuals to their fears. VR allows patients to discard possessions virtually, simulating the tactile experience of handling items and making decisions about them, bridging the gap between imagination and real-world scenarios [21]. Compared to imagined exposure, the sensory-motor engagement offered by VR may alleviate difficulties in envisioning stimuli and reduce the avoidance of triggering situations [16]. In recent years, there has been growing interest in applying VR as a therapeutic tool for various psychological disorders [22]. This preliminary study examines the effectiveness of non-immersive virtual environments in helping individuals from the general population discard possessions they find difficult to let go of. By focusing on a non-clinical sample, this research explores the potential of VR exposure as a motivational and behavioral intervention for hoarding tendencies [14]. By leveraging the unique advantages of non-immersive VR, this study aims to bridge the gap between immersive VR research and real-world applications, particularly for populations with limited access to costly and complex technologies. Incorporating VR into treatment strategies has the potential to improve patient engagement and motivation, as the technology caters to diverse learning preferences and delivers immediate feedback on progress in managing hoarding behaviors [14]. As research in this domain continues to advance, further studies are necessary to confirm the long-term effectiveness of VR interventions for HD [21]. Expanding on the existing body of knowledge, this study explores the effects of non-immersive VR environments on state anxiety, positive and negative emotional experiences, and the frequency of object discarding. To assess the emotional impact before and after the experience of discarding a personal item, it is crucial to examine both state anxiety and state feelings, as these psychological factors significantly shape emotional and behavioral reactions. State anxiety refers to a transient emotional state characterized by subjective feelings of tension and apprehension [23, 24]. Measuring state anxiety provides valuable insights into the immediate psychological effects triggered by object disposal, which can provoke discomfort or distress based on individual sensitivity. Recent work by Chasson et al. [25] has demonstrated the motivational benefits of VR for individuals with HD. In their pilot study, participants were exposed to clutter-free renderings of their own homes via VR, which led to increased readiness for treatment and greater confidence in change. These findings suggest that VR can help overcome treatment ambivalence by providing a compelling visual representation of a desired living environment, potentially increasing engagement in therapeutic interventions [25].

Likewise, state feelings encompass a broader range of emotional responses, capturing both positive and negative affective states. This expanded perspective offers a more holistic understanding of the psychological experience associated with exposure to discarding objects [26]. By addressing motivational and behavioral barriers, non-immersive VR enhances therapeutic outcomes, making interventions more engaging and less distressing for participants. Furthermore, St-Pierre-Delorme and O’Connor [27] have highlighted how non-immersive VR bridges the gap between cognitive-behavioral therapy and real-life applications in hoarding interventions. This research underscores the potential of non-immersive VR as a cost-effective, adaptable, and efficient alternative to traditional exposure methods for treating hoarding disorder and related conditions.

The present preliminary Randomized Controlled Trial (RCT) study aims to investigate whether exposure to a non-immersive VR environment can facilitate discarding personal belongings among individuals from the general population who desire to dispose of certain items but have difficulty doing so. An additional objective is to explore, through self-report instruments, the differences within and between subjects concerning various psychological constructs associated with, state anxiety, positive and negative feelings, and the frequency of discarding the identified object.

Given the limited and preliminary nature of existing evidence on the use of non-immersive VR in the context of discarding behaviors, particularly among individuals with hoarding tendencies in non-clinical populations, this study adopts an exploratory approach. While prior research suggests the potential benefits of VR for exposure-based interventions, there is currently insufficient empirical support to formulate strong, directional hypotheses specific to this modality and population. However, our predictions include:

### Hypothesis 1

Participants exposed to the non-immersive VR environment will be more likely to discard a personal object during or after the exposure session than participants in the imagination-based exposure group. This hypothesis is informed by preliminary findings indicating that non-immersive VR can simulate real-world behavior in a structured and emotionally manageable way, enhancing task engagement and facilitating behavioral change (e.g., [27]).

### Hypothesis 2

Both groups will report reductions in state anxiety and negative affect, and potential increases in positive affect, from pre- to post-exposure. The non-immersive VR group may exhibit greater emotional regulation due to the added structure and interactive features of the virtual environment. This is supported by findings that non-immersive VR exposure can reduce emotional reactivity and improve coping in various clinical contexts (e.g., [28, 29]).

## Methods

### Overview

The current study employed a Randomized Controlled Trial (RCT) to evaluate the effects of two exposure-based interventions, Virtual Reality Exposure (VRe) and Imagination Exposure (Ie), on individuals experiencing difficulty discarding possessions. Participants completed a one-time, in-lab exposure session following initial online screening and filled out a series of self-report questionnaires. Recruitment was conducted between March and October 2024. After providing informed consent and meeting eligibility criteria, participants were randomly assigned to one of the two experimental conditions. Randomization was conducted using a simple, computer-generated sequence via the “RAND” function in Microsoft Excel (Microsoft Corp), ensuring allocation concealment. The randomization was unrestricted, meaning no blocking or stratification procedures were employed. This approach ensured that each participant had an equal probability of assignment to either condition, regardless of enrollment order or participant characteristics. The random allocation sequence generation and participant enrollment were performed by two master’s-level psychology students. Blinding procedures were implemented to reduce bias. Although participants were aware of the intervention, they received due to the nature of the exposure tasks, outcome assessors were blinded to group allocation. Specifically, the researchers responsible for data analysis and interpretation were not involved in the delivery of the interventions and did not have access to identifying group information during scoring or analysis. This partial blinding was intended to minimize expectancy effects and reduce potential bias in outcome evaluation. Each participant completed pre- and post-exposure assessments measuring psychological and behavioral outcomes related to discarding behavior. The entire procedure consisted of one experimental session per participant. Each of these elements—recruitment, eligibility, group assignment, and session details—is described in more detail in the following sections. The trial was registered on the ClinicalTrials.gov website (ID NCT07160543).

### Participants and experimental design

Participants were recruited from a non-clinical convenience sample residing in the northeast of Italy, as the study required in-lab participation and easy physical access to the research facility. Recruitment was conducted through online messaging platforms and social networking websites, as well as in person during university lectures. Individuals interested in participating were invited to contact the research team via the email address provided in the recruitment materials. Participants had to meet the following inclusion criteria: (1) be at least 18 years old and have access to a computer or smartphone to complete online assessments; (2) score 4 or higher on the Hoarding Rating Scale item assessing difficulty in discarding possessions [26, 30]; (3) show no signs of suicidal ideation or clinical conditions that could interfere with participation (including psychotic disorders, major depressive disorders, substance abuse, cognitive impairments, or organic mental disorders); (4) report difficulty discarding at least one object; and (5) complete all online questionnaires and a custom object evaluation form. The selected object for the experimental task had to score in the intermediate range (4–6) on importance, difficulty discarding, and willingness to discard to ensure standardization across participants. These criteria, along with detailed screening and object selection procedures, are further described in the Procedure section.

#### Participant screening and exclusion

**Fig. 1:**
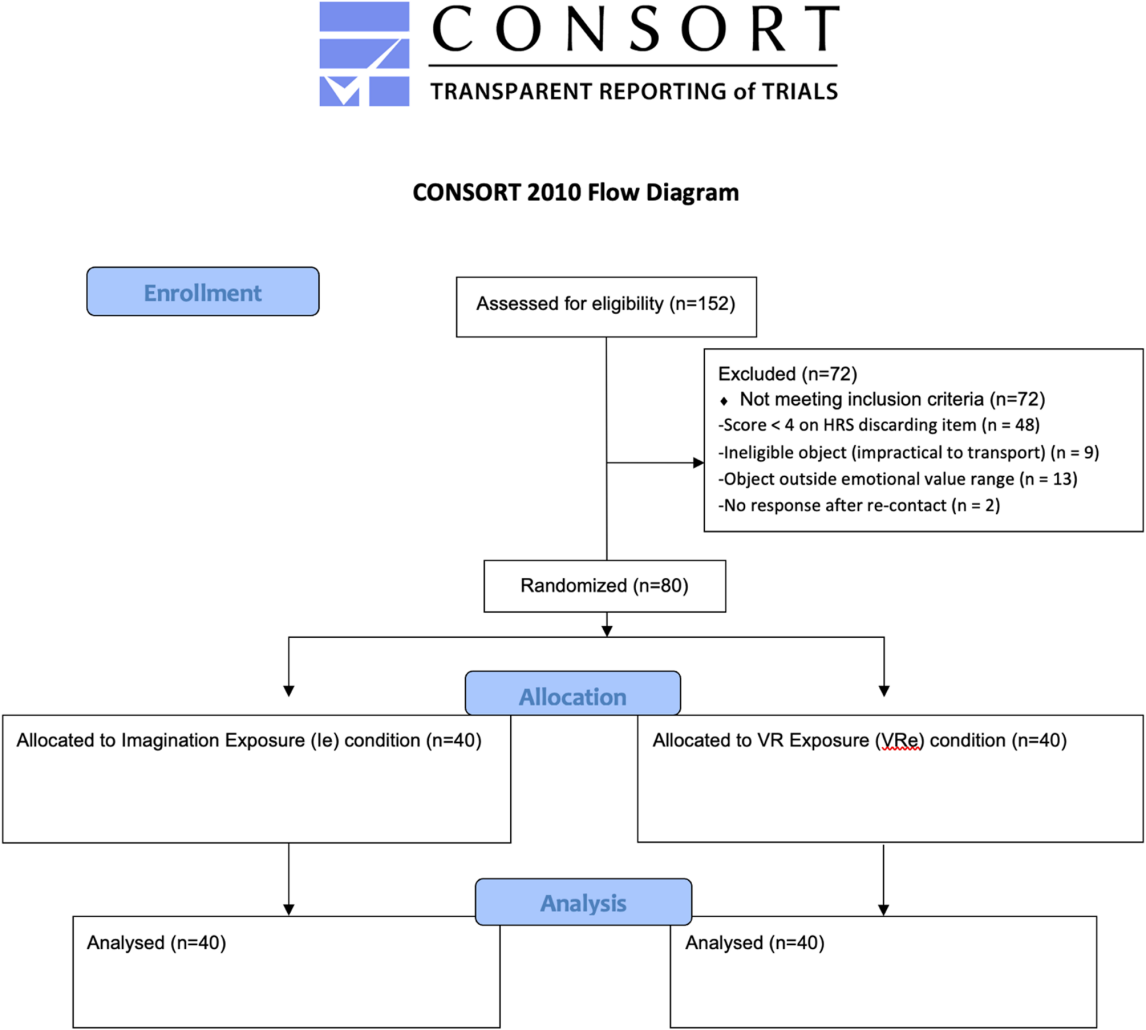
CONSORT Flow diagram

Of the total individuals who completed the initial screening questionnaire (*n* = 152), a substantial proportion (*n* = 72, 47.4%) did not meet the inclusion criteria and were therefore excluded from participation in the experimental session. The most frequent reasons for exclusion were: (1) a score below 4 on the self-report item of the Hoarding Rating Scale assessing difficulty discarding, indicating insufficient discarding-related difficulties; (2) ineligibility of the selected object, such as items deemed too important, emotionally extreme, or impractical to transport (e.g., smartphones, laptops, large items); (3) emotional value outside the inclusion range, where participants selected objects with sentimental value, but the item did not meet the required score range (4–6) across the three dimensions assessed (importance, difficulty discarding, and willingness to discard), thus compromising standardization; (4) non-response after re-contact, where individuals who initially met criteria failed to complete the follow-up steps (e.g., did not confirm their lab session or bring the object as instructed). In Fig. 1 are listed more detailed information regarding the reasons for participant exclusion.

In total, 80 individuals (52 women; 65%) were recruited, with a mean age of 25.98 years (SD: 9.84; min: 19, max: 62), and were randomly assigned to one of the following two conditions:

*Imagination exposure (Ie)*: In this condition, participants were asked to close their eyes and vividly imagine discarding the personal object they had previously selected. The procedure was guided verbally by the experimenter, who instructed participants to mentally visualize themselves moving the object from their personal space to a trash bin. The aim was to evoke the emotions and cognitive reactions associated with discarding in a controlled, internalized format. The session was conducted in a quiet room to minimize distractions, and the experimenter ensured participants remained focused on the visualization throughout the task. The guided imagery track was customized for each participant. Below is the transcription of the guided imagery track used during the Ie condition. This script was adapted for each participant based on the type of object they had selected and the personal content they shared. The aim was to ensure the visualization was as emotionally relevant and engaging as possible for each participant: *“Please make yourself comfortable*,* sit back*,* and gently close your eyes. Take a deep breath in… and out. Let your shoulders drop and allow your body to relax with each breath. Now*,* I’d like you to bring to mind the personal object you selected earlier. Picture it clearly in your mind [ its shape*,* texture*,* color*,* and any memories or thoughts associated with it]. Visualize yourself holding this object in your hands* [it depends on the type of object]. *Feel its weight* [it depends on the type of object]. *Notice any sensations that arise as you focus on it — physical feelings*,* emotional reactions*,* or thoughts. Now imagine your own personal space — the place where this object usually stays. See yourself standing in that space*,* holding the object*,* becoming aware of the decision you’re about to make. Slowly*,* picture yourself beginning to move. You’re walking through your home*,* carrying the object. Each step brings you closer to the bin — the place where you will leave this item behind* [In some cases, it was helpful to suggest that participants imagine bringing their own household trash bin to the location where the object was kept, in order to make the scenario more realistic]. *Now*,* see the bin in front of you. Take a moment to observe it. When you’re ready*,* imagine yourself placing the object inside the bin. Let your hand release it. See it fall and settle inside. Pause for a moment. What are you feeling now? Notice any thoughts*,* sensations*,* or emotional responses. Simply observe them without judgment. Now*,* begin to walk away from the bin. With each step*,* feel a sense of closure and distance from the object. Allow yourself to experience what it means to let go — whatever that looks like for you. Take one more deep breath in… and slowly release it. When you feel ready*,* gently bring your attention back to the room. Wiggle your fingers and toes. And when you’re ready*,* you may open your eyes”.*

*Virtual reality exposure (VRe)*: In this condition, participants interacted with a non-immersive, computer-based virtual environment displayed on a standard monitor. The environment simulated a household setting with various rooms and a trash bin located in a virtual garage. Participants navigated the space using a keyboard and mouse and were presented with a digital representation of their own personal object, embedded into the virtual scene. To ensure standardization and emotional congruence across participants, each individual was asked prior to the session to take a series of photographs of their selected item from multiple angles. These images were then integrated and adapted into the virtual environment, resulting in an exact visual representation of the real-life object brought to the lab. This procedure was designed to maximize the ecological validity of the task by enhancing identification with the virtual object and preserving its affective salience.

Participants were instructed to drag the object into the virtual trash bin, thereby simulating the act of discarding. The experimenter remained present to support and guide participants during the session. By engaging with a personalized and contextually relevant virtual scenario, this approach aimed to maintain the emotional relevance of the exposure and minimize variability in object valence across individuals. Following the VR interaction, all participants were asked whether they intended to discard the real object. They were then instructed to discard the same physical object in vivo, ensuring continuity between the virtual exposure and real-world behavioral outcome.

These two conditions serve as the independent variable for between-group comparison. Psychological constructs (e.g., state anxiety, positive/negative feelings) are measured at two points, before and after the exposure sessions. We assessed Psychological Constructs (State anxiety and Positive and Negative Affect) and Behavioral Outcomes (frequency of discarding the identified object during exposure and frequency of discarding during subsequent in vivo sessions). In Fig. 1 is the Consort Flow Diagram related to the main phases of the study.

### Software for the virtual environment development

An MSI PC was utilized for the VR session, and the Hoarding Disorder Test program was installed and developed internally at the Department of General Psychology of the University of Padua. Specifically, the Blender v.2.93 and the Godot v.3.2.3 software’s were used for the implementation of VR scenarios.

### Virtual reality environment

**Fig. 2:**
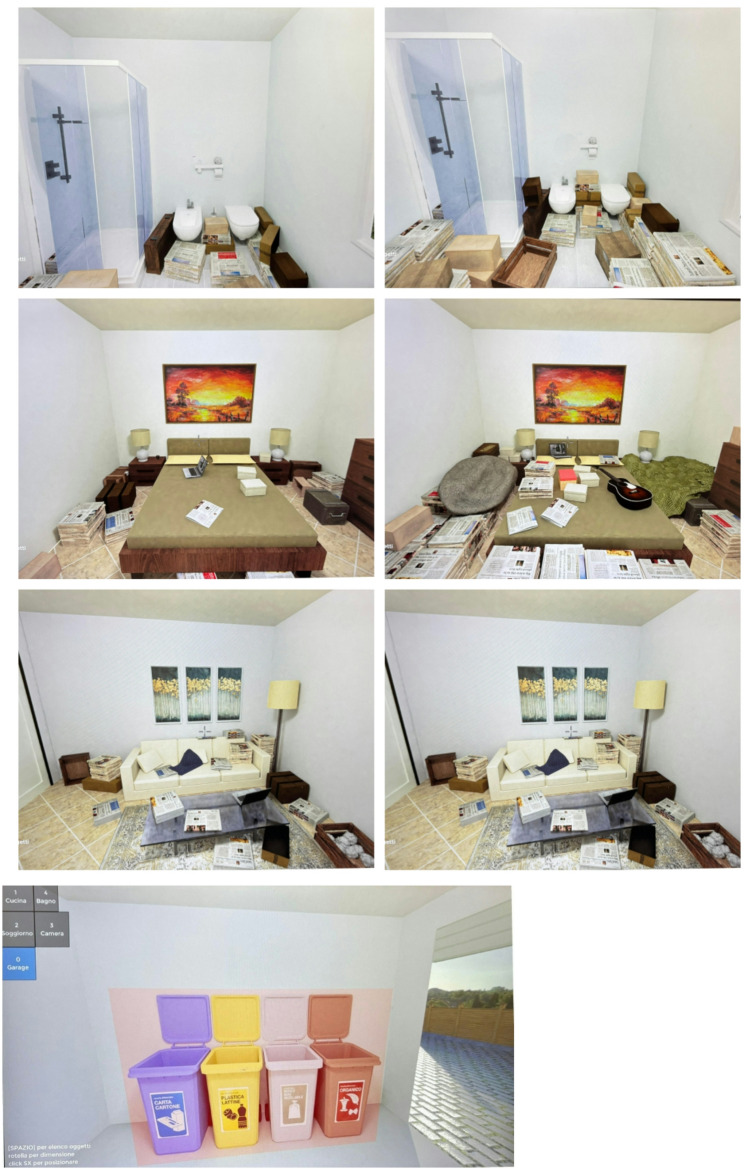
Examples of images taken from the non-immersive virtual scenario to which the participants of the VRe group were exposed

**Fig. 3:**
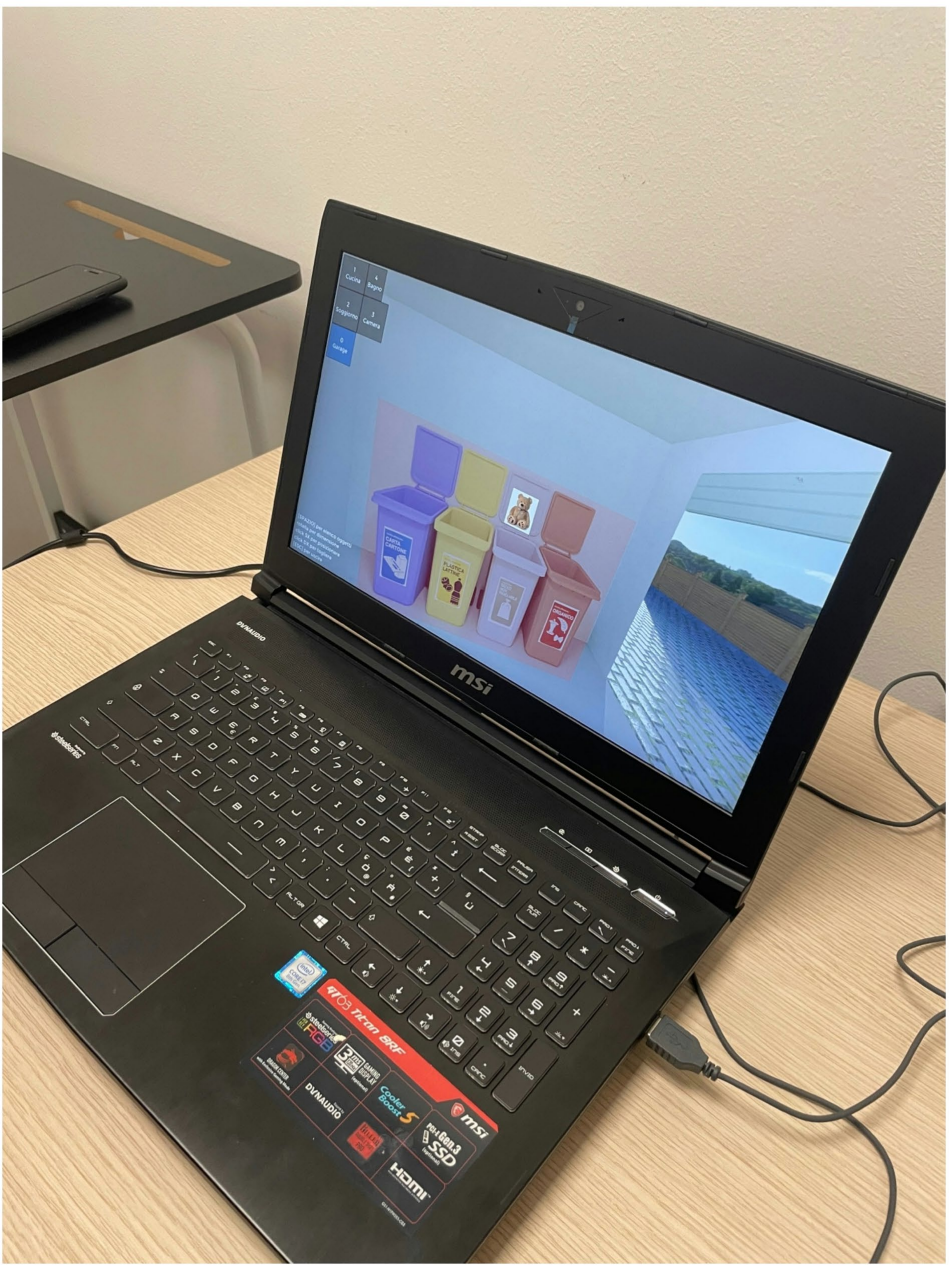
Participants were instructed to dispose of their object in a designated disposal area within the virtual environment, which featured various bins for object placement

The virtual environment is displayed on a PC monitor and includes images of key areas in a house (kitchen, bathroom, living room, bedroom, garage with trash bins). Using the “1-2-3-4-0” keys (1 = kitchen, 2 = bathroom, 3 = living room, 4 = bedroom, 0 = garage), participants can navigate between rooms and select and move various objects represented in the rooms using the mouse. To make the virtual contexts in which participants were exposed more realistic, participants were asked, before the VR experience, to indicate which level of clutter in the virtual environment best represented the state of the rooms in their home (Fig. 2). Participants were then asked to dispose of their object within the virtual context, and this was done in a specific area of the VR environment where various bins were displayed (Fig. 3).

### Measures

The following self-report questionnaires were administered:

The *Initial Recruitment Questionnaire*: Administered via the Qualtrics platform, this questionnaire included both demographic questions and screening items aligned with the study’s inclusion criteria. To be eligible, participants were required to: (1) be at least 18 years old; (2) have access to a computer or smartphone to complete the online questionnaires; (3) score 4 or higher on the self-report item of the Hoarding Rating Scale–Self Report [26, 30], which assesses difficulty discarding possessions; (4) show no evident signs of suicidal ideation based on the Beck Depression Inventory-II [31], and report no current alcohol or substance abuse, psychotic spectrum disorders, major depressive disorders, organic mental disorders, or cognitive impairment; and (5) report subjective difficulty discarding specific personal items. Additionally, participants were required to complete the initial monitoring questionnaires online via PC or smartphone.

#### The sociodemographic and screening questionnaire

Participants completed a self-report sociodemographic form designed to collect general background information, including age, gender, marital status, and level of education.

*The State-Trait Anxiety Inventory-Y* (STAI-Y; [23, 32, 33]): It is a self-report questionnaire divided into two scales (Y1 and Y2) that assess state and trait anxiety using the same items but with different and separate instructions. The tool consists of 40 items, and responses are evaluated based on a Likert scale distributed over 4 points (State Anxiety: from “not at all” to “very much”; Trait Anxiety: from “almost never” to “almost always”). The items related to state anxiety investigate the emotional activation associated with anxiety experienced at the moment the person is completing the test (e.g., “I feel calm,” “I feel secure,” “I am tense”). The items related to trait anxiety identify how the individual generally feels on a habitual basis (e.g., “I feel tense and restless,” “I am satisfied with myself,” “I have negative thoughts”). Pedrabissi and Santinello [28] adapted the Italian version of the questionnaire. In both versions, internal consistency is very high: the original version (20 items; 0.86 < α < 0.95) and the Italian version (20 items; 0.91 < α < 0.95). McDonald’s Omega was calculated for the total score, yielding a value of ω = 0.91. The value indicates excellent internal consistency since it exceeds the commonly accepted threshold of 0.70.

*The Positive and Negative Affect Schedule* (PANAS; [34, 35]): It is a self-report questionnaire aimed at investigating the dimensions of affective state, specifically Positive Affect (PA) and Negative Affect (NA) in individuals, both as stable traits and as contingent states. Positive affect is a construct that reflects the extent to which a person experiences pleasant emotions and states, such as excitement, enthusiasm, pleasurable engagement, and activity. Negative affect, on the other hand, encompasses a variety of adverse moods and refers to negative emotions and states, including anger, shame, guilt, fear, and nervousness.

The questionnaire consists of 20 items, 10 of which are related to positive affect (e.g., “active”), while the other 10 are related to negative affect (e.g., “nervous”). Responses are rated on a scale from 1 to 5, based on how the subjects feel at that specific moment, with 1 = “slightly,” 2 = “a little,” 3 = “moderately,” 4 = “quite,” and 5 = “extremely” [31]. In the Italian version [31], the item “concentrated” replaced “alert” because in Italian, “alert” has an ambiguous connotation, while “concentrated” has a clear positive meaning. Both versions show good internal consistency for the NA scale (α = 0.89) and the PA scale (α = 0.85) in the original version. The Italian version also demonstrates good internal consistency for the PA scale (10 items; α = 0.83) and the NA scale (10 items; α = 0.85). McDonald’s Omega was calculated for the PANAS-PA and the PANAS-NA total scores, yielding values of ω = 0.87 and ω = 0.91. The values indicate excellent internal consistency since they exceed the commonly accepted threshold of 0.70.

### Procedure

#### Initial assessment (T0)

**Fig. 4:**
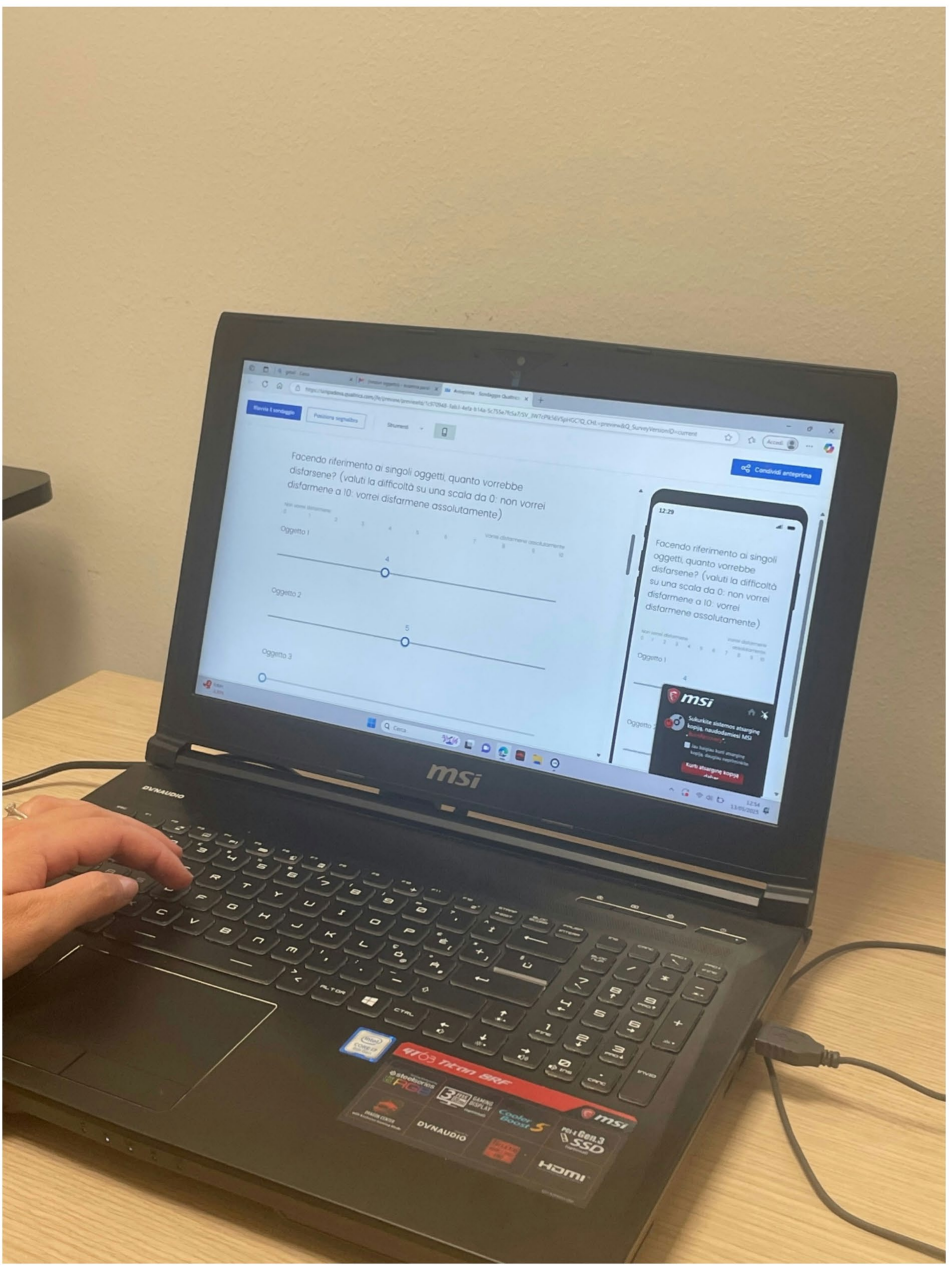
Participants completed a custom questionnaire in which they evaluated each object based on two dimensions: perceived importance and difficulty of discarding

Participant recruitment is deployed including announcements published on major social networks (e.g., (e.g., Facebook, WhatsApp, Instagram, Twitter), printed notices posted on bulletin boards in libraries and study rooms in Padua (Italy), and brief presentations of the research project during various courses at the University of Padua. Interested individuals were first directed to a Qualtrics survey, where they completed eligibility screening independently. A brief Zoom meeting was optionally offered to participants who wished to ask questions or receive further clarification about the study. These meetings did not include any screening procedures and served solely to provide information. In each case, contact information for the research team was provided in the recruitment materials to allow individuals to schedule such a meeting if desired. Subsequently, those who expressed their intention to participate in the study received an email containing a link to a questionnaire hosted on the Qualtrics platform. Before the questionnaires’ administration, participants signed a written informed consent form, based on Qualtrics platform, agreeing to participate in the study. They were informed that (1) their data would be confidential, (2) they could omit any information they did not wish to provide, and (3) they could withdraw from the study without explanation. Furthermore, all participants were required to be at least 18 years old, have access to a computer or smartphone for completing the online questionnaires, and meet the following inclusion criteria: (I) A score of 4 or higher on the self-report item of the Hoarding Rating Scale Self-Report [26], which assesses difficulty in discarding possessions; (II) No evident signs of suicidal ideation, based on the scores of the Beck Depression Inventory-II [35], nor indications of alcohol or substance abuse, psychotic spectrum disorders, depressive disorders, organic mental disorders, or conditions associated with cognitive impairment; (III) Participants must report difficulty in discarding certain objects. Moreover, individuals have to complete online monitoring questionnaires via PC or smartphone. Additionally, participants in this phase had to fill out a custom questionnaire evaluating each object based on two parameters: importance and difficulty in discarding (Fig. 4) (Table 1).

After completing the Initial Recruitment Questionnaire via the Qualtrics platform, individuals who met the inclusion criteria were re-contacted for the next phase of the experiment, which took place at the Experimental Psychopathology Laboratory (A-08) of the Department of General Psychology (Padua, Italy).

**Fig. 5:**
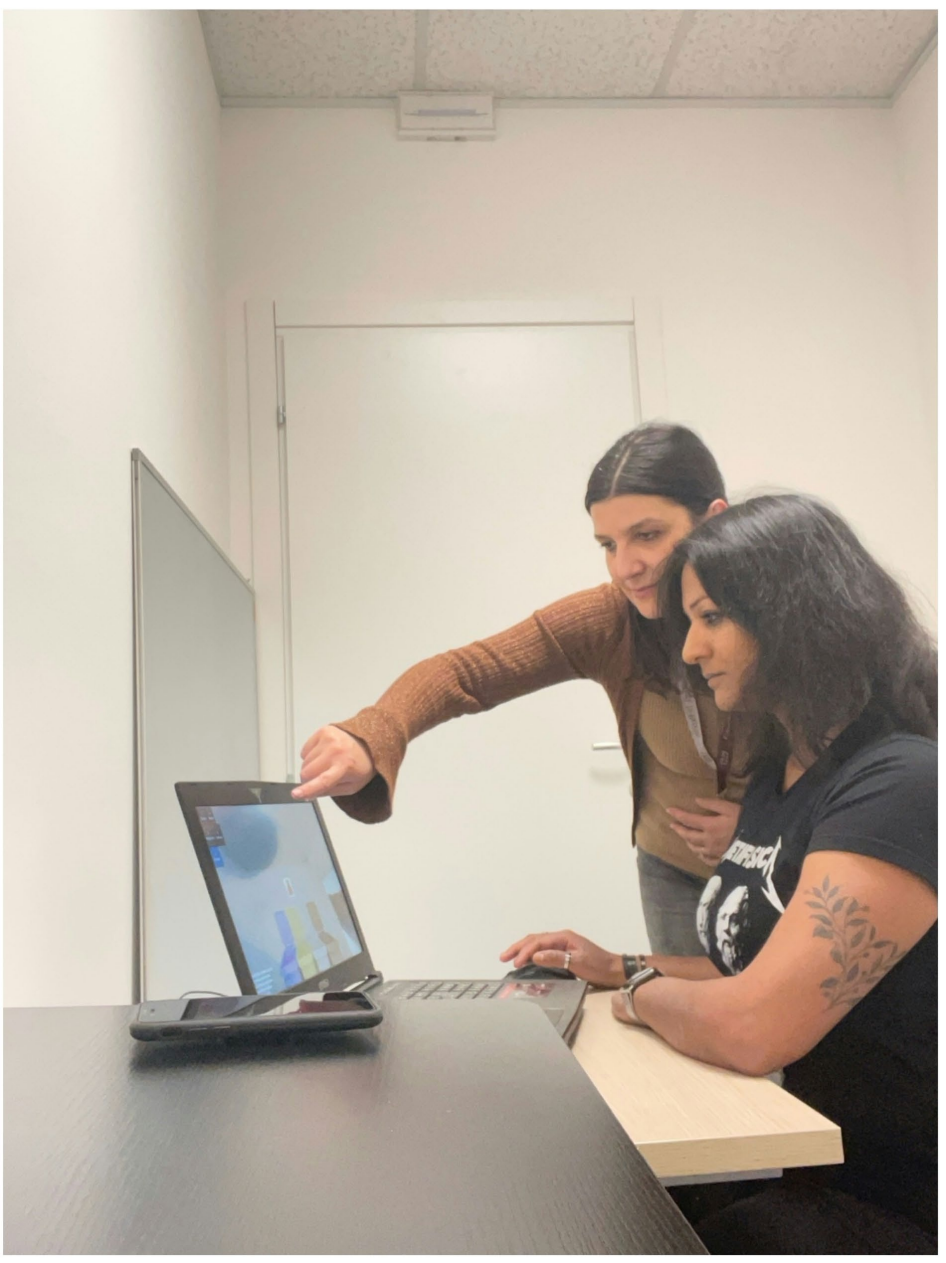
An experimenter supervised each phase of the laboratory procedure to ensure protocol adherence

Each phase of the laboratory procedure was supervised by one of the experimenters involved in the study (Fig. 5).

#### Object selection procedure

**Fig. 6:**
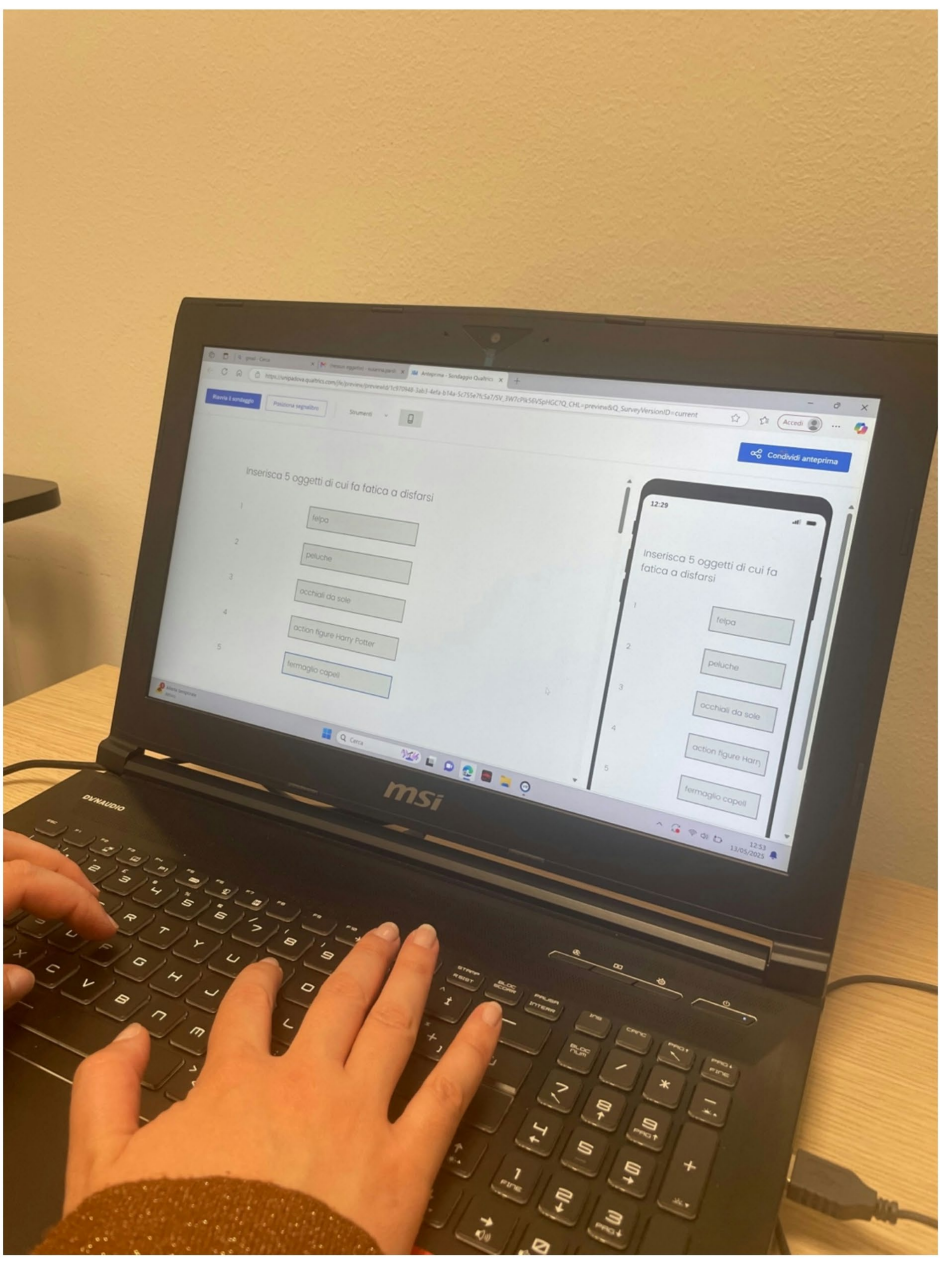
Participants completed a custom-designed questionnaire listing five personal items they found difficult to discard

To ensure consistency and comparability between groups, participants were asked to complete a custom-designed questionnaire in which they listed five personal items they found difficult to discard (Fig. 6; Table 1).

For each object, participants provided ratings on three 11-point Likert scales (ranging from 0 = not at all to 10 = extremely): perceived importance, difficulty in discarding, and willingness to discard. The object used in the experimental task was selected based on intermediate ratings (between 4 and 6) on all three scales, to minimize extreme emotional or practical attachments and ensure standardization across participants. Individuals were also asked to specify the type of value associated with each object. All participants reported that the selected object had sentimental value. For ethical and safety reasons, participants were instructed not to bring high-value or essential items such as smartphones, watches, or laptops. In addition to difficulty and transportability, selected objects also had to meet criteria related to emotional relevance (e.g., moderate levels of attachment and willingness to discard) and practical suitability for the study setting. These measures ensured that the task was both ethically appropriate and emotionally meaningful, while minimizing potential risks or undue distress. All participants were asked to bring the specified object to the laboratory on the day of the experiment.

Participants brought a wide variety of personal items, which were characterized by sentimental rather than material value. These objects were grouped into thematic categories based on their type and function: - Books and Written Materials (including schoolbooks, novels, elementary booklets, diaries, and notebooks); - Photographs and Letters (e.g., photo albums, framed photos, handwritten letters, and greeting cards); - Clothing and Accessories (e.g., sweatshirts, t-shirts, jeans, dresses, baby clothes, sports jerseys, scarves, bags, and shoes (both everyday and childhood-related); -Toys and Childhood Items (e.g., Disney characters, childhood toys, and action figures); - Souvenirs and Memorabilia (e.g., small souvenirs, jewelry, keepsake boxes, and religious icons); Miscellaneous Objects (e.g., a hockey uniform, a hair straightener, a toothbrush, and model collectibles (e.g., MotoGP).

Overall, the objects reflected a mix of everyday items and emotionally meaningful belongings, with the common thread being the participants’ reported difficulty in parting with them due to their affective significance.

**Fig. 7:**
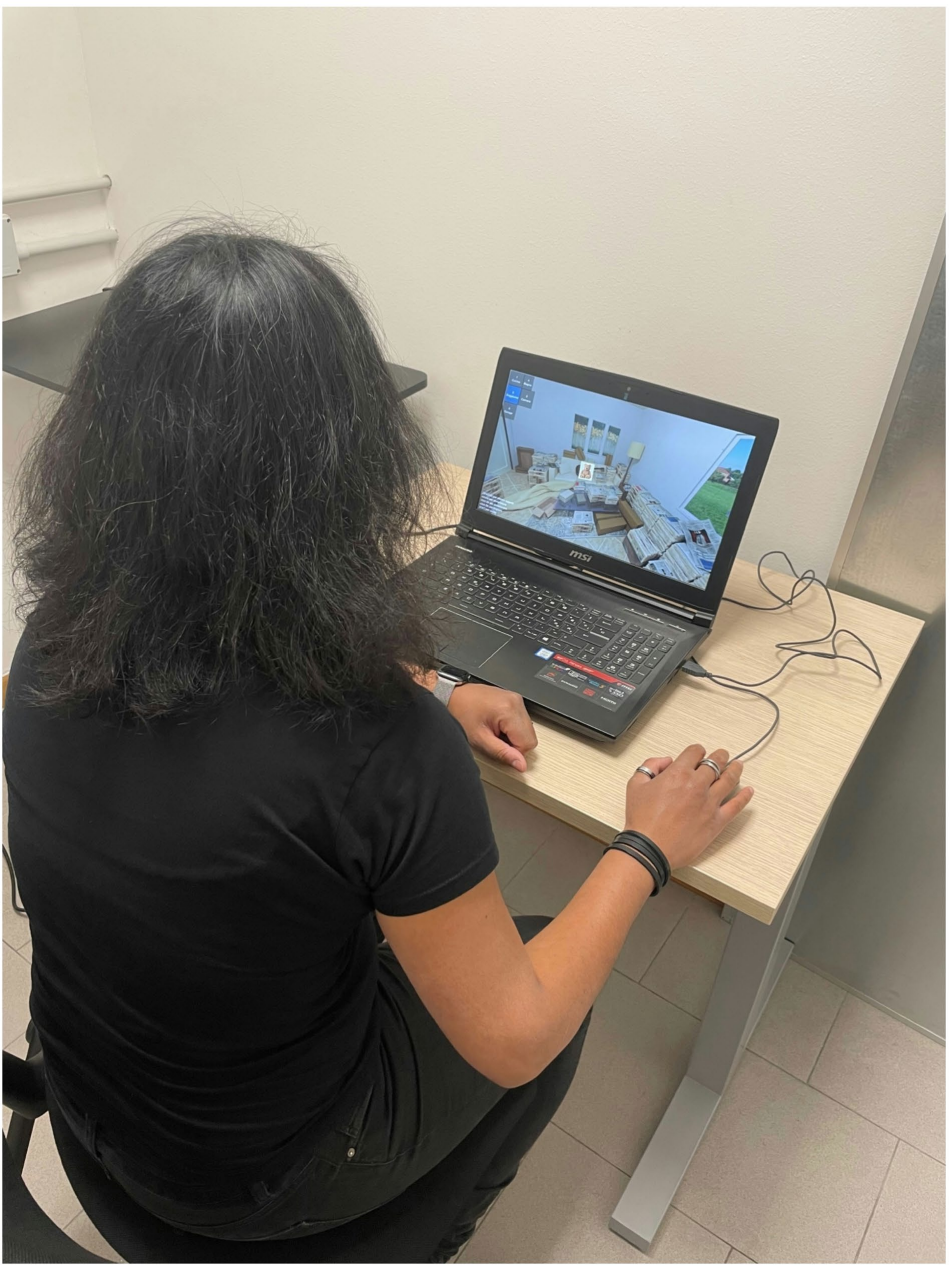
Example of a session conducted within the Virtual Reality environment (VRe)

- Pre-Exposure Assessment Phase (T1). Participants filled out the PANAS and the STAI-Y1 (Table 1).
- Exposure to the Experimental sessions (in VRe or in Ie and then in vivo) (Fig. 7; Table 1)
- Post-Exposure Assessment (T2). The questionnaires administered in phase T1 are repeated (the PANAS and the STAI-Y1). Phases T1, T2, and exposure will occur within the same session, with an expected total duration of approximately 45 min (Table 1).

**Table 1:** Summary of study phases

Phase	Description	Format and Location
T0 – Initial Assessment	Recruitment via social media, flyers, and lectures. Online screening through Qualtrics and Informed Consent Form.	Online (Qualtrics platform)
Object Evaluation	Participants rated 5 personal objects on importance, difficulty discarding, and willingness to discard. Object with intermediate scores selected.	Online (Ad hoc questionnaire)
T1 – Pre-Exposure Assessment	Participants completed the PANAS and the STAI-Y1 to assess emotional state prior to the exposure task.	In-lab (Qualtrics platform)
Exposure Task	Participants were randomly assigned to: • Virtual Reality Exposure (VRe) with their personal object embedded into a simulated environment. • Imagination Exposure (Ie) guided by experimenter. Both followed by in vivo discarding phase.	In-lab
T2 – Post-Exposure Assessment	Participants filled out the PANAS and the STAI-Y1 questionnaires to measure emotional changes after the task.	In-lab
Total Duration	Full session (T1, Exposure, T2) conducted in a single lab session. Estimated duration: ~45 min.	In-lab

### Statistical analysis

Quantitative analyses were conducted using SPSS (version 29; [36]). Descriptive statistics (frequencies, means, and standard deviations) were used to summarize participants’ sociodemographic characteristics. The internal consistency of the psychological scales (STAI-Y1 and PANAS) was assessed using McDonald’s Omega (ω), which offers a more robust estimate of reliability than Cronbach’s Alpha, particularly when item factor loadings are not uniform. To examine psychological outcomes, a 2 × 2 mixed factorial design was applied. This analysis involved two independent variables: Group (Virtual Reality Exposure [VRe] vs. Imagination Exposure [Ie]) as the between-subjects factor, and Time (pre-exposure [T1] vs. post-exposure [T2]) as the within-subjects factor. The dependent variables were state anxiety (as measured by the STAI-Y1) and positive and negative affect (as measured by the PANAS). These variables were analyzed using repeated-measures MANOVA. Where significant interactions or main effects were detected, follow-up ANCOVAs were performed to control for potential baseline differences, and pairwise comparisons were conducted using Least Significant Difference (equivalent to no adjustments). To evaluate behavioral outcomes, chi-square tests were employed. These analyses assessed between-group differences in the frequency of participants discarding the object during the exposure session and during the subsequent in vivo follow-up session. Behavioral responses were treated as categorical variables (discarded vs. not discarded).

## Results

### Demographics data

The current study involved two groups of individuals from general population, each composed of 40 participants: the Virtual Reality Exposure group (VRe) and the Imagination Exposure group (Ie). The Demographics data are resumed in Table 2. No significant differences emerged for all the demographics and clinical data (Table 2).

**Table 2:** Group means, standard deviations, and ANOVA results for demographics data, depressive symptoms, hoarding symptoms and the three dimensions assessed during the screening phase (Importance, difficulty discarding, and willingness to Discard) at baseline

Measure	Group(s)	*n*(%) or M(SD)	χ² or F_(1,78)_	*p*-value	η²ₚ
**Gender (Females%)**	VRe	25 (62.5%)	0.220	0.639	0.052
	Ie	27 (67.5%)			
**Age**	VRe	25.33 (8.83)	0.346	0.558	0.004
	Ie	26.63 (10.83)			
**Marital Status**	VRe	Single: 28 (70%)	2.064	0.559	0.162
		Married: 9 (22.5%)			
		Cohabiting: 3 (7.5%)			
		Divorced: 0			
	Ie	Single: 29 (74.4%)			
		Married: 8 (20.5%)			
		Cohabiting: 1 (2.6%)			
		Divorced: 1 (2.6%)			
**Educational Level**	VRe	High School Diploma: 20 (57.1%)	0.483	0.770	0.006
		Bachelor’s degree: 17 (48.6%)			
		Master’s degree: 1 (2.9%)			
	Ie	High School Diploma: 21 (52.5%)			
		Bachelor’s degree: 15 (37.5%)			
		Master’s degree: 2 (5%)			
**HRS-Total score**	VRe	12.38 (6.12)	0.001	0.973	0.000
	Ie	12.33 (4.57)			
**HRS-Clutter scale**	VRe	2.38 (1.98)	0.381	0.539	0.006
	Ie	2.09 (1.91)			
**HRS-Difficulty Discarding scale**	VRe	4.35 (1.64)	0.651	0.423	0.009
	Ie	4.64 (1.27)			
**HRS-Acquisition scale**	VRe	2.35 (1.53)	0.069	0.793	0.001
	Ie	2.45 (1.75)			
**HRS-Distress**	VRe	2.00 (1.62)	0.057	0.812	0.001
	Ie	2.09 (1.57)			
**HRS_5 – Impairment**	VRe	1.30 (1.51)	0.513	0.476	0.007
	Ie	1.06 (1.22)			
**Screening dimension 1 (Importance)**	VRe	4.90 (0.81)	0.020	0.522	0.000
	Ie	4.88 (0.76)			
**Screening dimension 2 (Difficulty Discarding)**	VRe	4.75 (0.54)	0.690	0.367	0.009
	Ie	4.85 (0.53)			
**Screening dimension 3 (Willingness to Discard)**	VRe	4.78 (0.42)	1.777	0.785	0.022
	Ie	4.93 (0.57)			
**BDI-II**	VRe	10.50 (10.45)	0.096	0.757	0.001
	Ie	11.20 (9.71)			

*Notes*: M = Mean; SD = Standard Deviation; VRe = Virtual Reality Exposure; Ie = Imagination Exposure; HRS = Hoarding Rating Scale; BDI-II = Beck Depression Inventory-II

### Differences related to psychological States

Firstly, we investigated for potential baseline differences between the two groups, based on a series of one-way ANOVAs on the screening dimensions considered (Importance, Difficulty Discarding, Willingness to Discard), as well as the total score and subscales of the Hoarding Rating Scale (HRS) and the Beck Depression Inventory II (BDI-II). Results indicated no significant group differences across any of these variables (Table 2). In light of these findings, none of these variables were included as covariates in subsequent analyses.

To examine changes in psychological states, a 2 × 2 repeated-measures MANOVA was conducted with Time (T1 vs. T2) as the within-subjects factor and Group (VRe vs. Ie) as the between-subjects factor. The dependent variables were state anxiety (STAI-Y1), positive affect (PANAS-PA), and negative affect (PANAS-NA). The analysis revealed no significant main effect of time (F_(3,76)_ = 1.64, *p-value* = 0.19, η²ₚ = 0.061) and no significant time × group interaction (F_(3,76)_ = 1.47, *p-value* = 0.23, η²ₚ = 0.055). A significant main effect of group emerged (F_(3,76)_ = 21.48, *p-value* < 0.001, η²ₚ = 0.459), indicating consistent between-group differences across both time points. At baseline (T1), participants in the Ie condition reported significantly higher state anxiety and negative affect compared to the VRe group (F_(1,78)_ = 47.56, *p-value* < 0.001, η²ₚ = 0.379). These differences remained directionally consistent at T2 (F_(1,78)_ = 63.04, *p-value* < 0.001, η²ₚ = 0.473). To further describe these differences, pairwise comparisons were conducted at T1 and T2 for each dependent variable. Results showed that participants in the VRe group reported significantly lower levels of state anxiety than those in the Ie group at both T1 and T2 (*p-value* < 0.001). Similarly, negative affect was lower in the VRe group at both time points (T1: F_(1,78)_ = 9.84, *p-value* = 0.004, η²ₚ = 0.112; T2: F_(1,78)_ = 8.73, *p-value* = 0.004, η²ₚ = 0.101). No significant between-group differences were found for positive affect at either time point (*p-value* = 0.37). These follow-up comparisons are presented for descriptive purposes only and should be interpreted with caution, as the lack of a significant interaction indicates that the pattern of change over time did not differ statistically between groups. In Table 3 are the Descriptive analysis for group differences and in Table 4 are the Between-Group Pairwise Comparisons at T1 and T2 for STAI and PANAS-NA and PANAS-PA scores. To complement the repeated-measures MANOVA and directly assess post-intervention group differences while controlling for baseline emotional states, a MANCOVA was conducted. Post-exposure scores of state anxiety (STAI-Y1), negative affect (PANAS-NA), and positive affect (PANAS-PA) were entered as dependent variables, with their corresponding pre-exposure scores used as covariates. The multivariate test revealed a significant effect of group (Wilks’ Λ = 0.720, F_(3,73)_ = 9.45, *p-value* < 0.001, partial η² = 0.280). Follow-up univariate analyses showed that, after adjusting for baseline values, participants in the VR group reported significantly lower levels of state anxiety (F_(1,75)_ = 27.41, *p-value* < 0.001, η² = 0.268) and negative affect (F_(1,75)_ = 0.37, *p-value* = 0.546), although the latter was not statistically significant. No between-group difference emerged for positive affect (F_(1,75)_ = 0.010, *p-value* = 0.919), (Table 5).

**Table 3:** Descriptive analysis for group differences

Measure(s)	Group(s)	M (SD)
**STAI-Y1 (T1)**	VRe	33.73 (7.34)
	Ie	43.05 (10.72)
**STAI-Y1 (T2)**	VRe	33.60 (5.92)
	Ie	46.45 (8.35)
**PANAS-NA (T1)**	VRe	13.70 (4.59)
	Ie	18.13 (8.34)
**PANAS-NA (T2)**	VRe	14.20 (4.30)
	Ie	18.65 (8.50)
**PANAS-PA (T1)**	VRe	28.40 (7.73)
	Ie	30.03 (8.49)
**PANAS- PA (T2)**	VRe	28.10 (8.04)
	Ie	28.55 (10.80)

*Notes*: M = Mean; SD = Standard Deviation; VRe = Virtual Reality Exposure; Ie = Imagination Exposure; STAI-Y1 = State-Trait Anxiety Inventory Form-Y1; PANAS-NA = Positive and Negative Affect Schedule - Negative Affect; PANAS-PA = Positive and Negative Affect Schedule - Positive Affect; T1 = Pre-Exposure Assessment Phase; T2 = Post-Exposure Assessment Phase

**Table 4:** Between-Group pairwise comparisons at T1 and T2 for STAI and PANAS scores

Measure(s)	Time of Assessment	(VRe) Group	(Ie) Group	MD (VRe-Ie)	SE	*p*-value
**STAI-Y1**	T1	VRe	Ie	-9.325*	2.055	< 0.001
	T2	VRe	Ie	-12.850*	1.619	< 0.001
**PANAS-NA**	T1	VRe	Ie	-4.425*	1.505	0.004
	T2	VRe	Ie	-4-450*	1.506	0.004
**PANAS-PA**	T1	VRe	Ie	-1.625	1.817	0.374
	T2	VRe	Ie	− 0.450	2.129	0.833

*Notes*: VRe = Virtual Reality Exposure; Ie = Imagination Exposure; MD = Mean Difference; SE = Standard Error; STAI-Y1 = State-Trait Anxiety Inventory Form-Y1; PANAS-NA = Positive and Negative Affect Schedule - Negative Affect; PANAS-PA = Positive and Negative Affect Schedule - Positive Affect; T1 = Pre-Exposure Assessment Phase; T2 = Post-Exposure Assessment Phase; * = The mean difference is significant al the 0.05 level

**Table 5:** MANCOVA results comparing post-exposure emotional outcomes between groups, controlling for baseline (T1) scores

Measure	Group(s)	M (SD)	F _(1,75)_	*p*-value	Partial η²ₚ
**STAI-Y1**	VRe	33.60 (5.92)	27.41	< 0.001	0.268
	Ie	46.45 (8.35)			
**PANAS-NA**	VRe	14.20 (4.30)	0.37	0.546	0.005
	Ie	18.65 (8.50)			
**PANAS-PA**	VRe	28.10 (8.04)	0.010	0.919	0.000
	Ie	28.55 (10.80)			

*Notes*: VRe = Virtual Reality Exposure; Ie = Imagination Exposure; M = Mean; SD = Standard Deviation; STAI-Y1 = State-Trait Anxiety Inventory Form-Y1; PANAS-NA = Positive and Negative Affect Schedule - Negative Affect; PANAS-PA = Positive and Negative Affect Schedule - Positive Affect

### Differences between groups about discarding possessions

**Fig. 8:**
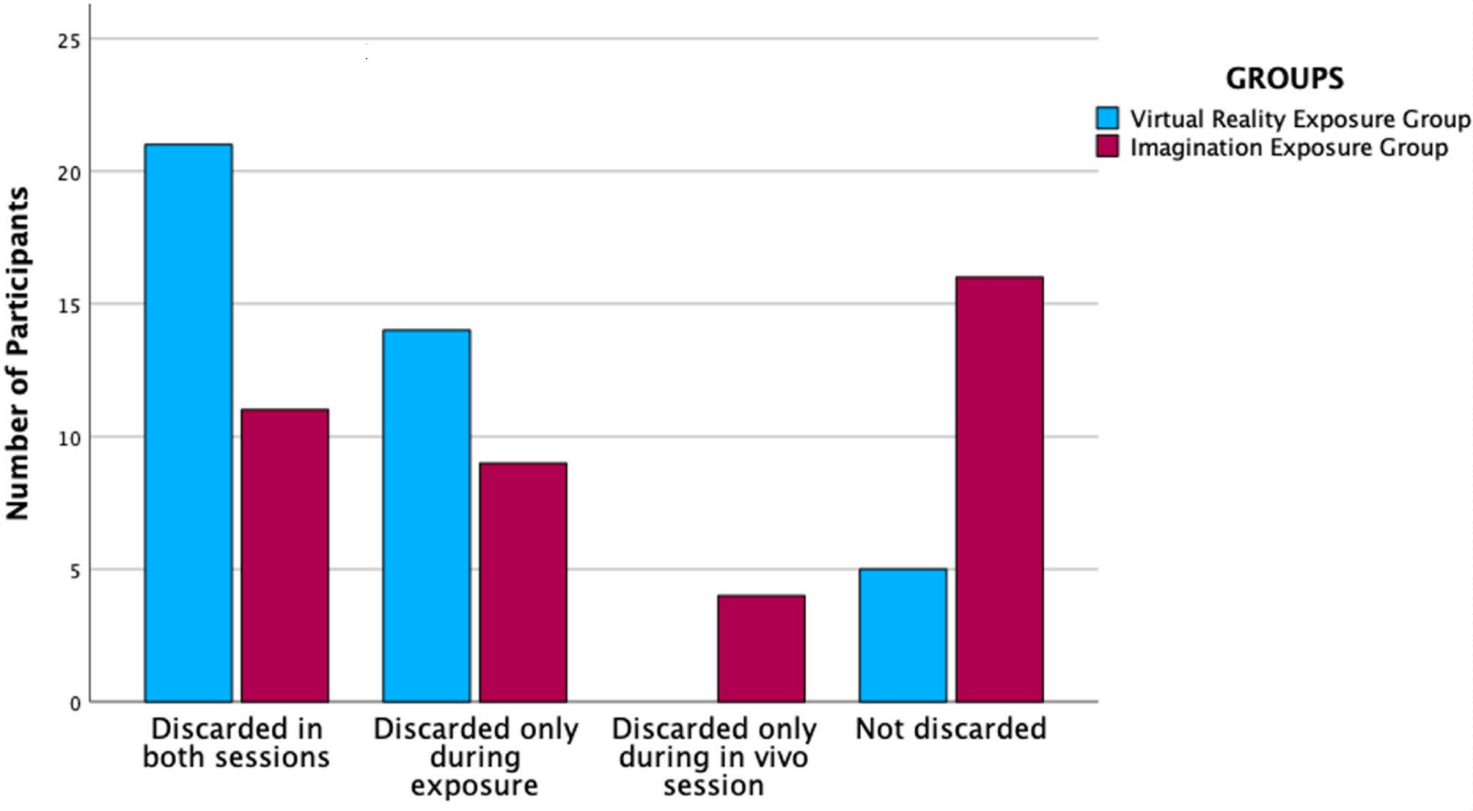
Comparisons between the groups regarding the frequency of throwing the object during the exposure session (in VR/in imagination) and during the in vivo session. *Notes*: *Discarded in both sessions*: it is referred to participants who discarded their object both during the experimental exposure session (either Virtual Reality or Imagination-based) and in the subsequent in vivo phase; *Discarded only during exposure*: it refers to participants who discarded the object exclusively during the experimental session but not in vivo; *Discarded only during in vivo session*: applies to participants who retained the object during the exposure but discarded it afterward in vivo; *Not discarded*: refers to participants who did not discard the object during either phase

A chi-square test revealed a significant association between group and object discarding during the experimental phase (χ²_(1, 80)_ = 13.53, *p-value* < 0.001) (Table 6). In the in vivo phase, the chi-square test showed no significant association between group and object discarding, χ²_(1,80)_ = 1.80, *p-value* = 0.179 (Table 6) (Fig. 8). To quantify group differences in object discarding behaviour, we computed odds ratios. During the experimental phase, participants in the VRe group were significantly more likely to discard the object compared to those in the Ie group (OR = 6.99, 95% CI [2.28, ∞], χ²(1) = 13.09, *p.value* < 0.001). In the in vivo phase, participants in the VRe group were 1.84 times more likely to discard the object than those in the Ie group; however, this difference did not reach statistical significance (OR = 1.84, 95% CI [0.75, 4.48], χ²(1) = 1.82, *p-value* = 0.178). Full results are presented in Table 6. These results suggest that group differences in object discarding behavior are primarily observed during the initial exposure phase, where participants in the VRe group were significantly more likely to discard objects compared to the Ie group.

**Table 6:** Discarded vs. Not discarded Objects – Experimental phase and in vivo phase

Phase	Group	Object Discarded	Object Not Discarded	Odds Ratio (95% CI)	χ²_(1)_	*p*-value
**Experimental**	**VRe**	35 (87.5%)	5 (12.5%)	**6.99** (2.28–21.70)	13.09	< 0.001
	**Ie**	20 (50%)	20 (50%)			
In Vivo	**VRe**	21 (52.5%)	19 (47.5%)	1.84 (0.75–4.48)	1.82	0.178
	**Ie**	15 (37.5%)	25 (62.5%)			

*Notes*: VRe = Virtual Reality Exposure; Ie = Imagination Exposure

A binary logistic regression was conducted to examine whether the experimental condition (VRe vs. Ie) predicted participants’ likelihood of discarding the object during the exposure session, while controlling for baseline state anxiety (STAI-Y1) and negative affect (PANAS-NA). The full model was statistically significant (Omnibus χ²(48) = 71.85, *p* = .014), suggesting that the predictors contributed to distinguishing between participants who discarded the object and those who did not. The model showed strong apparent fit (Nagelkerke R² = 0.833), good calibration (Hosmer–Lemeshow χ²(8) = 0.137, *p* = 1.000), and an overall classification accuracy of 91.3%. The experimental condition emerged as a significant predictor of discarding behavior: participants in the VRe group were significantly more likely to discard their object compared to those in the Ie group, even after adjusting for baseline emotional variables (Wald = 10.69, *p* = .001, Exp(B) = 2.20). Although STAI-Y1 and PANAS-NA did not emerge as statistically significant predictors, they were included in the model due to their theoretical relevance. Importantly, diagnostic checks conducted via linear regression indicated no problematic multicollinearity between these variables (both VIFs = 1.669; Tolerance = 0.599), justifying their concurrent inclusion. We have revised the language to clarify that the observed effect of the experimental condition was independent of, rather than controlled for, these emotional variables. These nuances, along with potential issues related to measurement or variable encoding, are discussed further in the Limitations section.

Finally, we tested whether discarding behavior during the experimental phase (VR or Ie) predicted real-life discarding decisions (in vivo phase). We ran a binary logistic regression with the in vivo discarding decision as the dependent variable and the experimental-phase decision as the predictor. The analysis revealed that discarding in the VR phase significantly predicted discarding in vivo, (B = 1.99, SE = 0.61, *p-value* = 0.001). The Odds Ratio (Exp(B) = 7.30, 95% CI [2.21, 24.15]) suggests that participants who discarded the item in the VRe phase were over 7 times more likely to discard it in real life. This finding indicates a strong link between simulated and real-world behavior, in line with the reviewer’s suggestion.

## Discussion

The present study preliminarily explored the effectiveness of non-immersive VR environments in facilitating the process of discarding objects among individuals with hoarding tendencies, compared to exposure through imagination. Additionally, it investigated within- and between-group differences on various psychological constructs, including state anxiety and positive and negative emotions experienced before and after the exposure to discard a personal object. The results support the use of VR as a behavioral tool in interventions targeting hoarding behaviors. A significantly higher proportion of participants in the VR group successfully discarded their object during the exposure compared to the imagination exposure group. These findings underline the potential of non-immersive VR as an effective behavioral intervention for hoarding tendencies. Outcomes align with previous research emphasizing the potential of VR to bridge the gap between exposure therapy and real-life behaviors by providing an immersive and interactive environment for practicing behavioral tasks [13, 14]. The findings also corroborate those of Delorme et al. [27], who demonstrated that non-immersive VR can effectively simulate real-world environments to enhance therapeutic outcomes in clinical populations compared to imagination. Importantly, no significant differences emerged between the groups when participants were asked to discard an object in vivo. This aspect warrants further investigation in future research to better understand the generalizability of VR-induced behavioral changes to real-world contexts. Similarly, prior studies have reported the utility of non-immersive VR in reducing anxiety-related responses in clinical contexts. For instance, Clemente et al. [28] observed positive outcomes using digital (non-immersive) stimulation in phobia treatment, supported by EEG and evoked potentials, while Cardi et al. [29] documented improvements in a patient with anorexia nervosa following a non-immersive VR-based intervention [28, 29]. These studies further reinforce the feasibility and psychological benefits of non-immersive VR interventions.

The current study also contributes to the growing literature on digital mental health tools for hoarding and related disorders. Recent studies have begun exploring the use of web-based platforms, cognitive bias modification, and other digital modalities to enhance engagement and reduce maladaptive behaviors. For example, David et al. [37] found that cognitive bias modification techniques reduced emotional attachment to possessions in individuals with high hoarding symptoms. Norberg et al. [38] tested an unguided web-based intervention aimed at reducing overconsumption, showing early promise for remote, scalable tools in managing compulsive acquisition. Similarly, Muroff et al. [39] piloted the Delivery of Internet Treatment for Compulsive Hoarding (DITCH) and demonstrated that internet-delivered interventions could improve treatment access and engagement. In a recent review, Hiranandani et al. [40] further emphasized the potential of digital mental health interventions for obsessive-compulsive and related disorders, calling for expanded innovation in delivery formats and personalization. Our findings extend this literature by highlighting how non-immersive VR may serve as a hybrid approach, offering the accessibility and scalability of digital interventions while maintaining a level of behavioral realism that facilitates experiential learning. Compared to traditional web-based programs or passive content delivery, VR allows users to practice discarding in immersive, dynamic environments. This may help bridge the gap between conceptual understanding and behavioral change, an area identified as a limitation in some digital interventions for hoarding and over acquisition.

The VR environment likely provided participants with a more engaging and tangible discarding experience, enhancing their ability to translate exposure into real-world behaviors.

Additionally, it is worth considering whether the option to select the clutter level in the virtual home may have influenced participants’ willingness to discard. This customizable feature may have increased self-awareness by prompting participants to reflect on their own real-life clutter levels, potentially heightening their motivation to engage with the task. Being visually confronted with a virtual environment that resembled their personal living conditions could have created a more emotionally salient context, fostering behavioral activation. Future research should investigate whether self-selected clutter intensity moderates discarding outcomes, as well as the emotional and motivational impact of such personalization features in virtual interventions.

Despite the significant behavioral differences, no substantial differences emerged within or between groups regarding state anxiety, positive emotional states, or negative emotional states, as assessed through the STAI-Y1 and PANAS scales. Follow-up pairwise comparisons indicated that participants in the VRe group had overall lower levels of state anxiety and negative affect than those in the Ie group at both time points, these findings should be interpreted with caution due to the absence of a significant time × group interaction and the lack of a main effect of time. Although we expected that the VRe condition would result in greater reductions in state anxiety and negative affect—based on prior literature suggesting that VR can reduce cognitive and emotional barriers to discarding—our findings did not reveal significant group differences on the PANAS or STAI-Y1. This outcome suggests that, while non-immersive VR may enhance behavioral engagement, its emotional impact may not differ substantially from that of imagination-based exposure in the short term. Several explanations are possible. First, both exposure types may have had comparable effects on emotional regulation, consistent with broader literature on exposure-based interventions. Another consideration is that the psychological measures employed may not have been sensitive enough to detect more nuanced emotional shifts following a single exposure session. Furthermore, emotional change in hoarding-related interventions may require repeated or more intensive sessions to fully emerge. These findings underscore the need to further investigate how different exposure formats influence both behavioral and emotional responses over time.

The present findings indicate that VRe was more effective than Ie in promoting object discarding behavior, particularly during the experimental phase. Participants in the VRe group were nearly seven times more likely to discard objects compared to those in the Ie group, a difference that was statistically significant and reflected a large effect size. This suggests that immersive environments may enhance emotional engagement and behavioral activation, thereby facilitating exposure-based outcomes in controlled settings. However, during the in vivo phase, although the VRe group still exhibited higher discarding rates than the Ie group, this difference was not statistically significant. This attenuation may reflect contextual or motivational barriers that arise when transitioning from structured experimental environments to real-life contexts. These findings highlight the potential of VRe as a powerful tool during the initial stages of exposure therapy, while also underscoring the need for strategies that support the generalization of learned behaviors into everyday settings. The enhanced behavioral outcomes observed in the VR group may be attributable to the realistic sensory-motor engagement provided by the virtual environment. Unlike imagined scenarios, VR offers participants a tangible and interactive platform to engage with their possessions, which likely reduces cognitive and emotional barriers to discarding objects. The use of a virtual home environment, complete with the representation of the participant’s object, may have also contributed to the increased ecological validity of the intervention. These findings highlight the potential of VR as a bridge between imagined and in vivo exposure, offering a safer and less emotionally taxing alternative for initial behavioral practice [16]. Non-immersive VR interventions may hold promise for other disorders characterized by avoidance behaviors, providing a scalable and effective tool for broader applications.

The results of the logistic regression analysis support the hypothesis that the mode of exposure influences discarding behavior. Participants in the VRe condition were more likely to discard their personal object compared to those in the Ie group, even when baseline emotional states were included in the model. This finding suggests that the virtual environment, which provided an interactive and visually grounded context, may have facilitated behavioral engagement with discarding tasks more effectively than guided imagery alone. Although initial differences in state anxiety and negative affect were present between groups, these variables did not emerge as reliable predictors in the model. Their inclusion, however, was theoretically grounded, as emotional states such as anxiety and negative affect are commonly associated with avoidance behaviors. To address potential concerns about multicollinearity, diagnostic analyses confirmed that these predictors were not collinear (VIFs = 1.669; Tolerance = 0.599), supporting their statistical independence and justifying their joint inclusion in the model. The lack of significance may instead reflect limitations in measurement sensitivity (e.g., reliance on baseline assessments), individual differences in affective processing, or the strong influence of the immersive manipulation, which may have overridden pre-existing mood states. Notably, even with baseline anxiety and negative affect included as covariates, the effect of exposure condition remained significant, the effect of exposure condition remained significant. Importantly, our analysis also revealed that discarding behavior during the experimental phase—regardless of whether it occurred in the VRe or Ie condition—was a strong predictor of real-life discarding behavior. Participants who discarded their item in the experimental phase were over seven times more likely to discard it in vivo, as indicated by the logistic regression model. This finding suggests that simulated discarding decisions, whether made in immersive virtual environments or through mental imagery, can meaningfully anticipate real-world behavioral outcomes. It reinforces the notion that simulated contexts serve not only as preparation but also as predictors of actual behavior. This is consistent with previous work on mental simulation and virtual environments [41, 42], and underscores the ecological and translational relevance of VRe-based interventions. From a therapeutic standpoint, this result highlights the utility of simulated environments not only for practicing difficult actions in a safer context but also for identifying individuals more likely to follow through with those actions in the real world. This highlights the relevance of the experimental manipulation itself, rather than emotional state, in predicting behavior. These findings reinforce the potential value of VRe in facilitating behavioral exposure tasks related to object discarding. The immersive and context-rich nature of VR may provide stronger cues for action and engagement than internally guided imagery alone. Future studies could benefit from repeated or dynamic assessments of emotional state (e.g., ecological momentary assessment), as well as alternative modeling approaches (e.g., latent variables or dimensional reduction) to further clarify the psychological mechanisms driving behavioral change in exposure contexts. Nonetheless, it is important to recognize that other affective or cognitive traits not assessed in the current study—such as depressive symptoms—could play a role in participants’ responses to exposure. Future research would benefit from a more comprehensive battery of baseline measures to better isolate the mechanisms underlying treatment effects. However, the lack of significant differences in psychological constructs between the groups raises important considerations. The measures used in this study were not sufficiently sensitive to detect nuanced differences from the intervention modalities. Alternatively, VR and imagination-based exposure may exert comparable effects on emotional constructs, emphasizing the importance of exposure therapy’s underlying principles rather than the delivery medium.

### Practical implications

While the present findings offer preliminary insight into the potential utility of non-immersive VR in facilitating discarding behavior, it is important to interpret these results within the context of a non-clinical convenience sample. The intervention tested in this study was not designed as a treatment for HD, but rather as an experimental paradigm to explore behavioral and emotional responses to discarding personal items. As such, conclusions regarding clinical efficacy should be made with caution. Nonetheless, the higher rates of object discarding observed in the VR condition suggest that non-immersive VR may serve as a useful tool in future interventions targeting hoarding-related behaviors, particularly for enhancing motivation and behavioral engagement. Future research should evaluate the feasibility, acceptability, and long-term effects of non-immersive VR exposure in clinical populations. Although not the focus of the present study, it is important to consider the implications of these findings for older adults with hoarding tendencies. Ayers et al. [43] showed that older adults with HD benefitted more from structured opportunities to practice discarding and making decisions about possessions in real or simulated environments, rather than relying solely on cognitive restructuring techniques based on the standard cognitive behavioral therapy. This preference reflects potential age-related differences in learning styles, motivation, and tolerance for abstract reasoning tasks, suggesting that therapeutic modalities which prioritize practical, hands-on engagement may be especially effective in this population. In light of these findings, non-immersive VR represents a particularly promising tool. It can offer controlled, repeatable, and low-risk behavioral exposure that closely simulates real-life discarding situations—an approach well-aligned with the intervention features that Ayers found to be most effective in older adults. For instance, a virtual environment can present older adults with realistic cues and contexts for practicing letting go of possessions, potentially increasing behavioral activation while reducing avoidance. However, while this modality holds promise, caution is warranted. Some older adults may experience difficulty navigating virtual environments due to unfamiliarity with technology, age-related sensory changes, or cognitive impairments. Studies have reported that certain VR features—such as complex interfaces, rapid visual transitions, or low usability—can lead to discomfort or disorientation in older populations. Thus, user-centered design and technological adaptation are critical if VR is to be successfully implemented in interventions for older adults with hoarding disorder. Features such as simplified navigation, larger text/icons, slower-paced transitions, and technical support could significantly improve accessibility and comfort. Future research should investigate the acceptability, feasibility, and effectiveness of VR-enhanced behavioral interventions in older adults, possibly incorporating findings from Ayers’ protocols, such as in-home discarding practice and structured exposure, to develop age-appropriate virtual experiences. These investigations could help clarify whether VR may serve as a valuable bridge between in-office therapy and real-world discarding practice for older individuals, while also addressing known treatment barriers in this population, such as mobility limitations or lack of social support. Moreover, future research should explore whether VR-based interventions can be adapted to meet the needs and preferences of older adults, and whether this modality can enhance engagement and outcomes in this population, especially when tailored to be cognitively accessible and user-friendly.

### Contribution to the literature

This study contributes to the limited but growing body of research on non-immersive VR by exploring its potential role in modifying behaviors associated with hoarding tendencies. While prior research has examined non-immersive VR in contexts such as phobia treatment [28] and anorexia nervosa [29], its application to hoarding-related behavior remains largely unexplored. Our findings are consistent with preliminary evidence suggesting that non-immersive VR can simulate real-world decision-making in a controlled environment and promote behavioral change [27]. By demonstrating that participants in the VR group were more likely to discard objects, this study supports the feasibility of using non-immersive VR as a scalable and low-cost alternative to immersive systems. It also highlights the potential for this modality to serve as a transitional step between imaginal exposure and in vivo behavioral interventions, offering new avenues for research and clinical practice in the treatment of hoarding-related behaviors.

### Limitations and future directions

This study has several limitations:

- Using a non-clinical convenience sample limits the generalizability of the findings to individuals formally diagnosed with HD. Future research should replicate these findings in clinical populations to validate the efficacy of non-immersive VR for HD.
- Although our sample size exceeds that of previous exploratory studies, the absence of an a priori power analysis and the exploratory nature of our research design limit the generalizability of our results. Future studies with larger, more diverse samples and rigorous power analyses are necessary to confirm and extend our findings.
- While the study utilized robust measures to assess state anxiety and emotional states, the lack of significant differences suggests the need for more targeted assessments, such as measures of avoidance behavior or subjective distress related to hoarding.
- The short duration of the intervention precludes conclusions about the long-term effects of VR-based exposure. Longitudinal studies are needed to evaluate whether the observed behavioral improvements translate into sustained reductions in hoarding symptoms.
- Another limitation of the present study lies in the absence of manipulation checks and the omission of measures capturing individual differences that may have influenced participants’ engagement and responses to the experimental conditions. Specifically, we did not verify whether participants in the VR condition experienced the non-immersive environment as realistic, nor whether those in the imagination condition were able to effectively visualize the exposure scenario. Additionally, we did not assess subjective perceptions of scenario quality, realism, or credibility, nor account for prior experience with virtual reality or guided imagery techniques—factors which may have moderated the interventions’ effectiveness. This lack of verification and individual-level profiling constrains the interpretability of the results and limits the conclusions that can be drawn regarding the underlying mechanisms of the observed effects. Future research should implement targeted manipulation checks and include assessments of relevant individual differences to ensure accurate interpretation of task engagement and perceptual validity across conditions.
- The study did not include attentional checks to confirm that participants remained focused during the experimental tasks. This absence limits our ability to ensure that responses reflect genuine engagement with the conditions rather than inattentive or distracted participation. Future studies should incorporate simple attentional control measures to verify that participants are actively involved throughout the procedure.
- An additional consideration concerns the possibility that participants may have begun to emotionally detach from their object prior to the experimental session. While this could influence discarding behavior, it is important to note that participants were not informed that they would be required to discard the object. The only instruction provided was to bring a personal item to the laboratory, without any mention of its potential disposal. Although some individuals may have speculated about the nature of the task—such as the possibility of discarding the object temporarily with the option to retrieve it—there was no explicit indication that permanent discarding would be expected. For this reason, we believe that any pre-exposure emotional disengagement was likely limited and not systematically driven by the study design. Future studies may benefit from assessing participants’ expectations prior to the session to further clarify this aspect.
- A key limitation of the present study concerns the modeling of covariates in the logistic regression analysis. Although baseline anxiety and negative affect were included based on strong theoretical grounds, they did not emerge as significant predictors of discarding behavior. Diagnostic checks ruled out multicollinearity between these variables (VIFs = 1.669; Tolerance = 0.599), suggesting that their instability may be due to other factors—such as limited measurement sensitivity, timing of assessment (pre-exposure only), or individual differences in affective response. Moreover, the study did not include additional psychological covariates (e.g., depressive symptoms) that may influence discarding tendencies. Future studies should incorporate a broader range of affective and cognitive variables and ensure adequate statistical power to reliably model their effects.
- A methodological limitation of the present study concerns the high rate of participant exclusion following the initial screening phase. Many individuals were excluded due to low levels of discarding difficulty or because their selected objects did not meet the standardized emotional relevance criteria (i.e., scores outside the 4–6 range on importance, difficulty discarding, and willingness to discard). While this rigorous selection process ensured experimental control and comparability across participants, it also highlights a challenge inherent in recruiting non-clinical samples for studies on discarding behavior—namely, the limited availability of individuals whose difficulties fall within a meaningful yet ethically manageable range. Furthermore, the high number of dropouts due to practical or motivational issues (e.g., failure to bring the object) underscores the logistical barriers associated with in vivo paradigms. Future research would benefit from methodological refinements that streamline object selection and eligibility assessment, potentially through automated pre-screening tools or hybrid inclusion models. Importantly, these findings also reinforce the need to extend this line of research to clinical populations, where the relevance and severity of discarding-related distress is greater. Studying individuals with diagnosed hoarding disorder or related conditions could provide more ecologically valid data, increase generalizability to clinical settings, and help determine whether VR-based interventions offer distinct advantages in populations with heightened emotional reactivity and behavioral avoidance.

Future studies could also investigate the intensity and type of emotions experienced in relation to the act of discarding the object in the experimental condition (in VR or through imagination) as well as after physically discarding the object. This would allow for a deeper exploration of the emotional state experienced by participants under these conditions.

Lastly, while this study highlighted the utility of non-immersive VR, the relative advantages of immersive versus non-immersive systems remain uncertain. Future research should directly compare these modalities to identify the optimal level of immersion necessary for the effective treatment of HD. Additionally, incorporating a follow-up measurement could provide valuable insights into the long-term sustainability of both behavioral and psychological changes induced by the interventions. To further enhance the study’s internal validity, it would be beneficial to include a third group, such as a no-intervention or waitlist control group, to better isolate the effects of VR-based treatments.

## Conclusion

The results of this preliminary study suggest that non-immersive VR environments offer a promising approach to enhancing exposure-based interventions for hoarding behaviors. VR may help individuals overcome the emotional and cognitive barriers associated with HD by providing an interactive and realistic platform for practicing discarding tasks. While further research is needed to validate these findings and explore their applicability to clinical populations, this study highlights the potential of VR as an innovative and accessible tool for addressing the complex challenges of hoarding disorder.

## Supplementary Information

Below is the link to the electronic supplementary material.

Supplementary Material 1

## Data Availability

Data will be available upon reasonable request to the corresponding author who has full access to the data reported in the manuscript.

## Abbreviations

HD: Hoarding disorder
CBT: Cognitive-behavioral therapy
VR: Virtual reality
VRe: Virtual reality exposure
Ie: Imagination exposure
STAI-Y1: State-trait anxiety inventory-Y1
PANAS: Positive and negative affect schedule
PA: Positive affect
NA: Negative affect
RCT: Randomized controlled trial
ANOVA: Analysis of variance
SPSS: Statistical package for the social sciences
